# Gluten-free diet affects fecal small non-coding RNA profiles and microbiome composition in celiac disease supporting a host-gut microbiota crosstalk

**DOI:** 10.1080/19490976.2023.2172955

**Published:** 2023-02-07

**Authors:** Antonio Francavilla, Giulio Ferrero, Barbara Pardini, Sonia Tarallo, Laura Zanatto, Gian Paolo Caviglia, Sabina Sieri, Sara Grioni, Giulia Francescato, Francesco Stalla, Cristina Guiotto, Lucia Crocella, Marco Astegiano, Mauro Bruno, Pier Luigi Calvo, Paolo Vineis, Davide Giuseppe Ribaldone, Alessio Naccarati

**Affiliations:** a Molecular and Genetic Epidemiology, Italian Institute for Genomic Medicine (IIGM), Torino, Italy; b Department of Computer Sciences, University of Torino, Torino, Italy; c Department of Clinical and Biological Sciences, University of Torino, Torino, Italy; d Institut d’Investigació Biomèdiques August Pi i Sunyer (IDIBAPS), Barcelona, Spain; e Division of Gastroenterology, Department of Medical Sciences, University of Torino, Torino, Italy; f Epidemiology and Prevention Unit, Fondazione IRCCS Istituto Nazionale dei Tumori di Milano, Milano, Italy; g Gastroenterology and Digestive Endoscopy Unit, “Città della Salute e della Scienza” Hospital, Torino, Italy; h Gastroenterology, Hospital Mauriziano Umberto I, Torino, Italy; i Pediatric Gastroenterology Unit, Department of Pediatrics, Azienda Ospedaliero-Universitaria Città della Salute e della Scienza di, Torino, Italy; j School of Public Health, Imperial College London, London, UK

**Keywords:** Stool microRNAs, gut microbiota, celiac disease, gluten-free diet

## Abstract

Current treatment for celiac disease (CD) is adhering to a gluten-free diet (GFD), although its long-term molecular effects are still undescribed. New molecular features detectable in stool may improve and facilitate noninvasive clinical management of CD. For this purpose, fecal small non-coding RNAs (sncRNAs) and gut microbiome profiles were concomitantly explored in CD subjects in relation to strict (or not) GFD adherence over time. In this observational study, we performed small RNA and shotgun metagenomic sequencing in stool from 63 treated CD (tCD) and 3 untreated subjects as well as 66 sex- and age-matched healthy controls. tCD included 51 individuals on strict GFD and with negative transglutaminase (TG) serology (tCD-TG-) and 12 symptomatic with not strict/short-time of GFD adherence and positive TG serology (tCD-TG+). Samples from additional 40 healthy adult individuals and a cohort of 19 untreated pediatric CD subjects and 19 sex/age matched controls were analyzed to further test the outcomes. Several miRNA and microbial profiles were altered in tCD subjects (adj. p < .05). Findings were validated in the external group of adult controls. In tCD-TG-, GFD duration correlated with five miRNA levels (p < .05): for miR-4533-3p and miR-2681-3p, the longer the diet adherence, the less the expression differed from controls. tCD-TG+ and untreated pediatric CD patients showed a similar miRNA dysregulation. Immune-response, trans-membrane transport and cell death pathways were enriched in targets of identified miRNAs. *Bifidobacterium longum, Ruminococcus bicirculans*, and *Haemophilus parainfluenzae* abundances shifted (adj. p < .05) with a progressive reduction of denitrification pathways with GFD length. Integrative analysis highlighted 121 miRNA-bacterial relationships (adj. p < .05). Specific molecular patterns in stool characterize CD subjects, reflecting either the long-term GFD effects or the gut inflammatory status, in case of a not strict/short-time adherence. Our findings suggest novel host-microbial interplays and could help the discovery of biomarkers for GFD monitoring over time.

## Introduction

Celiac disease (CD) is a complex autoimmune disease occurring in ~1% of the population worldwide.^1^ Gluten ingestion triggers an autoimmune reaction leading to duodenal damage characterized by villous atrophy and resulting malabsorption.^2^ CD diagnosis in adults is based on the positivity of serum tissue-transglutaminase 2 (TG) IgA antibodies, confirmed by histopathological analyses of duodenal biopsy.^3,4^ However, this diagnostic approach presents some limitations, such as the low capacity to accurately test CD in seronegative subjects or the fact that villous atrophy can also be due to medical treatments or other enteropathies besides CD.^5^ So far, the only treatment for CD is to adhere to a lifelong gluten-free diet (GFD), although its long-term effects have not been thoroughly investigated. Thus, novel biomarkers for CD diagnosis and monitoring of the GFD adherence/effects could be helpful in the management of this disease.^6^

The immune system dysregulation or the GFD-imposed dietary regime could lead to transcriptional/post-transcriptional alterations potentially involving small non-coding RNAs (sncRNAs), key cellular regulators of gene expression.^7^ microRNAs (miRNAs) are ~22-nt-long molecules targeting genes involved in pivotal cellular processes and diseases, including those of the gastrointestinal tract.^8,9^ Although they are the most studied class of sncRNAs, few studies have characterized miRNA expression in CD, performing the analyses in intestinal epithelium and blood only.^10–12^

Accumulating evidence associated specific nutrient intake with miRNA expression.^13,14^ In particular, our group previously reported that fecal miRNA profiles change with anthropometric traits and lifestyle habits^15^ as well as accurately distinguish subjects with different dietary habits.^16^ In this respect, miRNAs could also reflect the impact of the GFD on CD subjects. Furthermore, diet modulates the gut microbiome composition and dietary regimes rich in fibers and poor in animal-based products drive a selection of specific taxa.^17^ In parallel, many studies have associated gut microbiome dysbiosis and several gastrointestinal disorders, including CD.^18^ Intriguingly, there is evidence of mutual regulation between fecal miRNAs and host gut microbiota mediated by diet-induced microbial metabolites.^19–21^ All these findings suggest that the diet-intestinal microbiota-miRNA axis could be crucial for regulating the host gene expression.^22^ However, the effect of GFD on this regulatory axis is still unexplored.

In the present study, we analyzed stool miRNA and other sncRNA profiles as well as the gut microbiome composition in treated CD (tCD) individuals on GFD. The aim was to explore specific patterns related to a strict dietary regime (with negative TG serology) or a not strict/short time adherence to GFD (with related gastrointestinal symptoms and positive TG serology). In addition, we also investigated whether the GFD length impacts the profiles of host and microbial investigated molecular features. Finally, we integrated outcomes from small RNA, shotgun metagenomic sequencing, and food/nutrient intake to explore host-gut microbial crosstalk in relation to GFD.

## Patients and Materials and methods

### Study population

One hundred and thirty-two subjects were enrolled by the Italian Institute for Genomic Medicine (IIGM), the Gastroenterology Unit of Ospedale Mauriziano Umberto I and the Gastroenterology outpatient clinic of San Giovanni Antica Sede, all in Turin, Italy. The cohort included tCD (n = 63), untreated CD (i.e., recruited before starting the GFD; n = 3), and healthy volunteers with no dietary restrictions (n = 66). For all participants, the inclusion criteria were as follows: no use of antibiotics and other drugs in the month before sampling, age >18, and no concomitant gastrointestinal disorders (e.g., allergies/intolerances, diabetes, inflammatory bowel diseases, or tumors). For CD subjects, besides the aforementioned criteria, a histological confirmation of the disease diagnosis was also required. The TG IgA levels were measured in serum samples of all subjects who provided blood for the present study, unless not already clinically tested in concomitance with the recruitment.

All participants signed an informed consent to participate in the study and received two questionnaires from the European Prospective Investigation into Cancer and Nutrition (EPIC) study (one on dietary habits and another, sex-specific, about lifestyle habits)^23^ and a disposable container for stool collection.

To validate the relevant differences in stool miRNA profiles/microbiome composition, small RNA-sequencing/metagenome data from an independent group of 40 healthy omnivorous adult individuals were compared with those of the study population hereby recruited. This cohort is fully described by Tarallo et al.^16^ Finally, additional small RNA-sequencing data from 38 pediatric subjects were included in this study to compare miRNA profiles observed in untreated CD/tCD-TG+ adult subjects. This set of patients was recruited within an ongoing research study in collaboration with the Gastroenterology Unit of the Regina Margherita Children’s Hospital (Torino, Italy). The group included children between 3 and 14 years old with a new diagnosis of CD (n = 19), untreated, and sex/age-matched with controls (n = 19). The inclusion criteria for these subjects were as follows: 1) negative medical history of concomitant or previous gastrointestinal diseases (including tumors) and 2) serological and histological verification/exclusion of the CD diagnosis.

The study was conducted according to the Declaration of Helsinki and approved by the local Ethics committees (Azienda Ospedaliera-Universitaria, Città della Salute e della Scienza di Torino, Protocols n.0030717 and n.0085713).

### Dietary information

The EPIC Food Frequency Questionnaire (FFQ) was used to determine the daily intake of foods/beverages whose nutrient composition was obtained from the Italian food composition tables.^24^ The composition of 24 gluten-containing foods listed in the FFQ was modified for the celiac population to include the recipes of composite or generic gluten-free foods.^25^

### Stool RNA and DNA isolation

The sample collection is described in Supplementary Materials. RNA was extracted from 200 µl aliquots using stool total RNA purification kit (Norgen Biotek Corp.) following manufacturer’s protocol. RNA quality and quantity were verified following the MIQE guidelines (http://miqe.gene-quantification.info/). The RNA concentration was quantified with the Qubit microRNA assay kit (Invitrogen). The DNA was extracted with the DNeasy PowerSoil Pro Kit (Qiagen) following manufacturer’s instructions with a higher final elution buffer volume (50 µl) to increase DNA concentration which was quantified with Qubit DNA high-sensitivity assay kit (Invitrogen).

### Small RNA-sequencing

Small RNA transcripts were converted into barcoded cDNA libraries using the NEBNext multiplex small RNA library prep set for Illumina (New England BioLabs), starting with 250 ng of RNA for each sample. The libraries were sequenced on Illumina HiSeq 4000 (Illumina) at the Gene Core Facility, Heidelberg (Germany). The complete procedure is described in^26^ and in Supplementary Materials. Raw small RNA-sequencing data were deposited at Gene Expression Omnibus with the identifier GSE217915.

### Shotgun metagenomics

Metagenomic sequencing of DNA from stool was done using the Illumina DNA Prep kit (Illumina) described in^27^ and performed on the NovaSeq 6000 Sequencing System (Illumina). Read pre-processing and alignment on PhiX control, as well as human genome were performed as in the study by Thomas et al.^27^ and fully described in Supplementary Materials. Raw sequencing data were deposited in the Sequence Read Archive (SRA) with the identifier PRJNA904924.

### Statistical and computational analyses

Statistical analyses were performed with R v4.0.4. Age and sex-adjusted differential expression analysis was performed with DESeq2 v1.22.2 (LRT function),^28^ adjusting for multiple testing by Benjamini–Hochberg method. Correlation analyses were performed using the Spearman’s rank correlation coefficient (SCC) and the analysis of monotonic miRNA expression trend using the Mann–Kendall test.

DIABLO module of MixOmics v.6.14.1 was used to integrate microbial profiles, differentially expressed miRNA levels, and dietary information,^29^ while classification analysis for the feature selection was performed with Weka v.3.8.5 with the CfsSubsetEval module. A detailed description is provided in Supplementary Materials.

## Results

### Population characteristics

**Figure 1.**
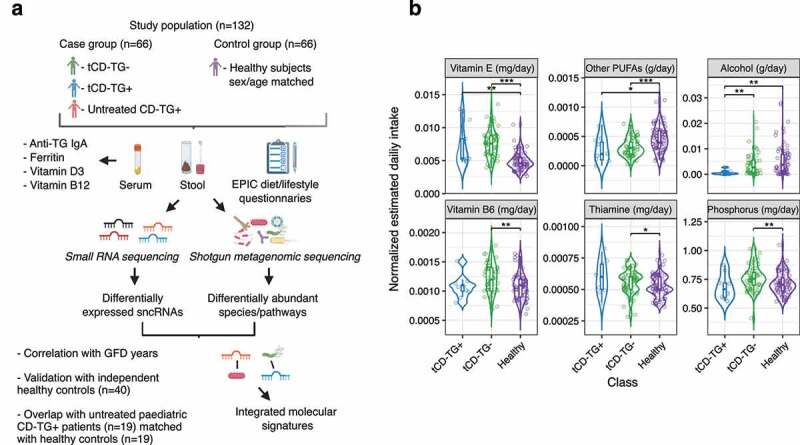
a) Workflow of the study. b) Violin plots reporting the estimated daily intake of nutrients significantly different among the investigated groups. The nutrient intake was normalized on the daily intake of kilocalories estimated for each subject. Wilcoxon Rank-Sum test: *p < .05; **p < .01; ***p < .001; tCD, treated Celiac Disease; TG, transglutaminase 2; GFD, gluten-free diet; sncRNAs, small non-coding RNAs.

In this study, 63 tCD, 3 untreated CD individuals, and 66 sex- and age-matched healthy controls, all adults and without specific dietary restrictions, were recruited (Figure 1a, Table 1, and Supplementary Table S1a).

**Table 1.** Characteristics of the study population.

Covariates		Untreated CD (n = 3)	tCD (n = 63)	Healthy controls (n = 66)	P-value tCD vs Healthy
Age (years)	Average ± SD Range	46.5 ± 13.9 31–58	42 ± 14.5 19–76	40.8 ± 14.3 20–78	0.78
Sex	Female Male	3 0	48 15	51 15	0.80
BMI (kg/m^2^)	Average ± SD	n.a.	22.3 ± 3.4	22.5 ± 3.1	0.21
Duration of Gluten-free diet (years)	Average ± SD Range	_	8.9 ± 7.9 2mo.-30	_	
Smoking status	current former never n.a.	1 1 1 0	10 15 37 1	10 15 41 0	0.99
Marsh grade	I II IIIa IIIb IIIc n.a.	0 0 1 2 0 0	5 2 15 13 16 12		
*s-Ab anti transglutaminase lgA (AU/ml)	≤3.0 >3.0 n.a.	0 3 0	51 12 0	64 0 2	
**s-Ferritin (ng/ml)	Average ± SD Range <11 ≥11 n.a.	6 ± 2 4–8 3 0 0	62.4 ± 62.3 8–246 4 46 13	59.1 ± 65.8 3–429 4 55 7	0.55
***s-Vitamin B12 (pg/ml)	Average ± SD Range <180 ≥180 n.a.	369 369 0 1 2	270.4 ± 108.9 111–708 7 41 15	273.4 ± 134.6 101–1152 11 48 7	0.55
****s-Vitamin D3 (ng/ml)	Average ± SD Range <30 ≥30 n.a.	17.8 17.8 1 0 2	61.1 ± 108.2 10–428 24 24 15	39.9 ± 70.7 3.8–92.7 47 12 7	6.19E-04

* Ab anti transglutaminase lgA serum test is negative when ≤3 AU/ml, dubious when between 3–12.0 AU/ml, and positive if >12 AU/ml.

** Ferritin serum level is low when <11 ng/ml, normal when ranging from 11 to 307 ng/ml, and high if >307 ng/ml.

***Vitamin B12 serum level is low when <180 pg/ml, normal when ranging from 180 to 914 pg/ml, and high if >914 pg/ml.

****Vitamin D3 serum level is low when <30 ng/ml, normal when ranging from 30 to 100 ng/ml, and high if >100 ng/ml.

n.a.: not available

The mean age of tCD subjects was 42.0 ± 14.5 years, and there was a prevalence of females (75%) (Table 1). No significant differences in BMI and smoking habits were observed between tCD subjects and healthy controls. The mean serological levels of vitamin D3 were higher in tCD individuals (61.1 ± 108.2 ng/ml) compared to controls (39.9 ± 70.8 ng/ml, p < .0001) due to the use of vitamin supplements, as reported in the questionnaires by one-third of tCD subjects (data not shown).

Fifty-one tCD individuals were on a strict GFD and tested negative for TG IgA antibodies (hereafter defined as tCD-TG-) at recruitment. The remaining 12 tCD subjects, presenting gastrointestinal symptoms at recruitment or starting the GFD in close proximity to it (i.e., from 1 to 3 months) or declaring to not strictly follow an appropriate dietary regime, all tested positive for TG IgA antibodies (hereafter defined as tCD-TG+). The three untreated CD subjects were also characterized by high levels of TG IgA antibodies (untreated CD-TG+).

The weekly intake of specific foods/drinks and the estimated daily nutrient intake are summarized in Supplementary Table S1b. Vitamin E intake (mg/day) was higher in tCD-TG- and tCD-TG+ subjects than in controls (p < .001 and p < .01, respectively), while intake of polyunsaturated fatty acids (PUFAs) other than linoleic, linolenic, and oleic acids resulted lower in the same comparisons (p < .001 and p < .05, respectively) (Figure 1b and Supplementary Table S1b). A higher intake of vitamin B6, thiamine, and phosphorus characterized the tCD-TG- group with respect to controls (p < .01), while alcohol consumption was lower in tCD-TG+ with respect to both the other study groups (p < .01).

### Stool miRNA profiles

An average of 122,382 small RNA-sequencing reads were aligned to human miRNome with an average of 756 miRNAs detected per sample (Supplementary Table S2a).

**Figure 2.**
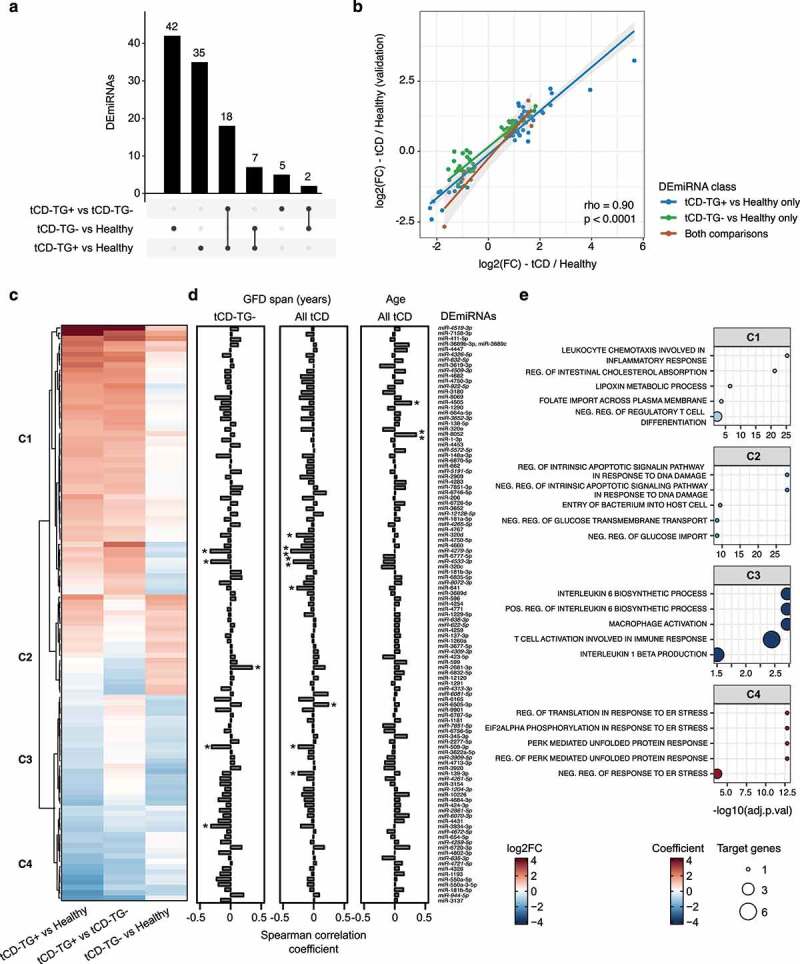
a) Upset plot reporting the number of differentially expressed miRNAs (DEmiRNAs) in each comparison performed among the study groups. b) Scatterplot relating the DEmiRNA log2 Fold Changes (log2FCs) from the comparison between the tCD groups and age- and sex-matched healthy controls (x-axis) or an independent group of healthy controls (y-axis) from.^16^ The color code represents the comparison in which each DEmiRNA was detected. c) Heat map of log2FCs of the DEmiRNAs in the comparison groups described in the study. c1 to c4 labels depict four clusters from the hierarchical clustering analysis. d) Correlation analyses between DEmiRNA levels and years of gluten-free diet (GFD) in tCD-TG- (left) and all tCD (middle), or subjects’ age (right). *p < .05; **p < .01. e) Dot plots showing the five most representative Gene Ontology Biological Processes enriched in the functional analysis of the DEmiRNAs belonging to each of the clusters reported in panel c. The dot size is proportional to the significance, while the color code refers to the RBiomirGS coefficient. Negative and positive coefficients are related to processes predicted to be, respectively down- and up-regulated based on the miRNA expression change.

An age- and sex-adjusted miRNA differential expression analysis was performed among tCD-TG-, tCD-TG+, and controls. In total, 109 differentially expressed miRNAs (DEmiRNAs) were identified (Figure 2a and Supplementary Table S3a), with 44 in tCD-TG- (17 up- and 27 down-regulated) and 53 in tCD-TG+ (39 up- and 14 down-regulated) compared to controls. Seven DEmiRNAs were dysregulated in both tCD groups with respect to controls. Finally, comparing tCD-TG+ and tCD-TG-, 25 DEmiRNAs (2 down- and 23 up-regulated in tCD-TG+) were identified (Figure 2a).

The profiles of the 109 DEmiRNAs observed in the tCD groups were compared with those of an independent cohort of 40 healthy omnivores (average age 40.5 ± 13.2, 24 females and 16 males, Supplementary Table S1a) analyzed in our lab,^16^ reporting a coherent differential expression (SCC = 0.90, p < .001) and confirming 77 DEmiRNAs (Figure 2b and Supplementary Table S3b). Five miRNAs (*miR-944-5p*, miR-3934-3p, miR-1229-5p, *miR-4672-5p*, and miR-4447) out of the seven identified as differentially expressed in both tCD groups when compared to healthy individuals were confirmed as significantly altered also in this second comparison. In contrast, in the individual categories, 28 and 37 miRNAs were confirmed in tCD-TG- and tCD-TG+, respectively.

Clustering analysis based on the expression changes of the 109 DEmiRNAs highlighted four distinct sets: 51 specifically up-regulated miRNAs in tCD-TG+ (cluster 1), 18 and 22 miRNAs, respectively, up- (cluster 2) and down-regulated (cluster 3) in both tCD groups, and 18 miRNAs down-regulated in tCD-TG+ (cluster 4) (Figure 2c). The differences in expression of miRNAs belonging to cluster 1 and cluster 4 were reduced between tCD-TG- and healthy subjects, corresponding to similar levels in these groups. In particular, 21 of these miRNAs showed an opposite expression trend as the one observed for the tCD-TG+ when compared with healthy subjects.

### Stool tCD-TG- DEmiRNA profiles in relation to GFD length

To further explore the relationship between miRNA dysregulation and GFD length, a correlation analysis was performed between DEmiRNA levels in tCD-TG- subjects and the years of GFD. The analysis highlighted five miRNAs that were significantly related to the length of the dietary regime: miR-3934-3p (SCC = −0.32), *miR-4279-3p* (SCC = −0.37), *miR-4533-3p* (SCC = −0.32), and miR-509-3p (SCC = −0.25), as negatively correlated, and miR-2681-3p as positively (SCC = 0.35) (Figure 2d and Supplementary Table S3c). The levels of these miRNAs were not concomitantly related to subjects’ age (Figure 2d and Supplementary Table S3c). After stratification of tCD-TG- subjects according to different GFD length categories, the differences in miRNA levels between tCD-TG- and healthy controls were reduced or increased with increasing years of adherence to the diet for some miRNAs as *miR-4533-3p* and miR-2681-3p (Supplementary Figure S1a).

### Profiles of tCD-TG+ DEmiRNAs in untreated CD-TG+ subjects

To evaluate whether the DEmiRNA dysregulation observed in tCD-TG+ could be mirrored in the active form of untreated CD, we were able to recruit only three CD-TG+ adult subjects before they started the GFD. Since this number of individuals was not statistically sufficient for robust differential expression analyses, the data from these subjects were merged with those from tCD-TG+ and the comparison with healthy controls repeated. The outcomes of the analysis confirmed 57 of the 60 DEmiRNAs in this comparison, including the seven DEmiRNAs in overlap with the tCD-TG-. Among the confirmed DEmiRNAs, nine showed a significant trend of expression (p < .05) going from untreated CD-TG+, tCD-TG+, and tCD-TG- to controls, with three progressively increasing and six decreasing (Supplementary Figure 1B and Supplementary Table S3c).

Additionally, given the limited number of untreated CD-TG+ in the cohort, we explored stool miRNA profiles from small RNA-sequencing data of an ongoing study on untreated pediatric CD patients (n = 19, average age = 10.4 ± 3.1, 9 females and 10 males, Supplementary Table S1a) matched for sex and age with healthy controls (n = 19, average age = 9.9 ± 3.2). Thirty-two (53%) of the 60 DEmiRNAs observed in the adult tCD-TG+ were characterized by a coherent expression with those of the pediatric CD-TG+ subjects (23 up-regulated and 9 down-regulated) compared to healthy controls (SCC = 0.68, p < .001) (Supplementary Figure S1c). Three out of the 32 coherently altered miRNAs were also dysregulated (p < .05) in the group of untreated CD-TG+ children, including miR-7158-3p, one of the nine miRNAs associated with a significant expression trend from adult untreated CD-TG+ to healthy subjects (Supplementary Table S3d and Supplementary Figure S1c,d).

### miRNA target enrichment analysis

The functional analysis of the four identified clusters of DEmiRNAs (Figure 2c) highlighted 372 enriched biological processes that were merged in 45 groups based on their semantic similarity (adj. p < .05, Supplementary Table S4a). Specifically, cluster 1 DEmiRNAs were mainly associated with inflammatory pathways (i.e., *leukocyte chemotaxis involved in inflammatory response, negative regulation of translation in response to stress, negative regulation of regulatory T cell differentiation*), intestinal absorption, and transmembrane transport pathways (i.e., *regulation of intestinal cholesterol absorption, folate import across plasma membrane*). Cluster 2 DEmiRNAs were enriched in terms related to apoptosis (i.e., *regulation of intrinsic apoptotic signaling pathway in response to DNA damage*, or *intrinsic apoptotic signaling pathway, negative regulation of intrinsic apoptotic signaling pathway*) and glucose transport (i.e., *negative regulation of glucose import, negative regulation of glucose transmembrane transport*), while cluster 3 DEmiRNAs were enriched in pathways connected to immune response (i.e., *positive regulation of interleukin 6 biosynthetic process, T cell activation involved in immune response, macrophage activation, interleukin 1β production)*. Finally, cluster 4 DEmiRNAs were mainly enriched in endoplasmic reticulum stress (i.e., *regulation of translation in response to endoplasmic reticulum stress* or *negative regulation of response to endoplasmic reticulum stress*).

Investigating the individual miRNA-target annotations, miR-6756-5p/miR-4447 targeting *DDX39B* was supported by the highest number of evidence (n = 48), followed by miR-181b-5p-*HSPA1B* (n = 20), miR-148a-3p/miR-139-3p targeting *HLA-A*, miR-6835-5p-*DDX39B*, and miR-7851-3p-*MICB* interactions (both with n = 16) (Supplementary Table S4b,c).

### Other fecal sncRNA profiles

An average of 416,834 reads were assigned to sncRNA annotations other than miRNAs, with an average of 2,356 detected sncRNAs (Supplementary Table S2a). Sixteen sncRNAs were significantly up-regulated in tCD-TG+ vs controls (Supplementary Table S5a and Supplementary Figure S2), including 11 piRNAs and 5 tRNAs. All 16 sncRNAs were also up-regulated in tCD-TG+ vs tCD-TG- (piR-43815 being statistically significant). The expression levels of the 16 DEsncRNAs up-regulated in tCD-TG+ were validated in the independent group, with five of them significant (piR-58832, piR-47969, piR-57921, tRNA-Phe (GAA), and piR-34680) (Supplementary Table S5b).

### Stool microbial profiles

An average of 44,458,589 metagenomic sequencing reads were generated for each sample (Supplementary Table S2b). Overall, 105 microbial species were detected on average in each sample and no significant differences emerged comparing the population heterogeneity (α-diversity) (Supplementary Table S6a). At phylum level, a substantial reduction of Actinobacteria (log2 fold change (log2FC) = 1.38; p < .001), Verrucomicrobia (log2FC = −1.17; p < .01) and an increase in Bacteroidetes (log2FC = 0.11; p < .05) abundances were reported in tCD-TG- with respect to controls (Supplementary Table S6b). The tCD-TG+ group showed a reduction of Euryarchaeota (log2FC = −5.46; p < .05) and an increase of Fusobacteria (log2FC = 0.24; p < .05) abundance in comparison with controls, while no differences between both tCD groups were observed (Supplementary Table S6b).

**Figure 3.**
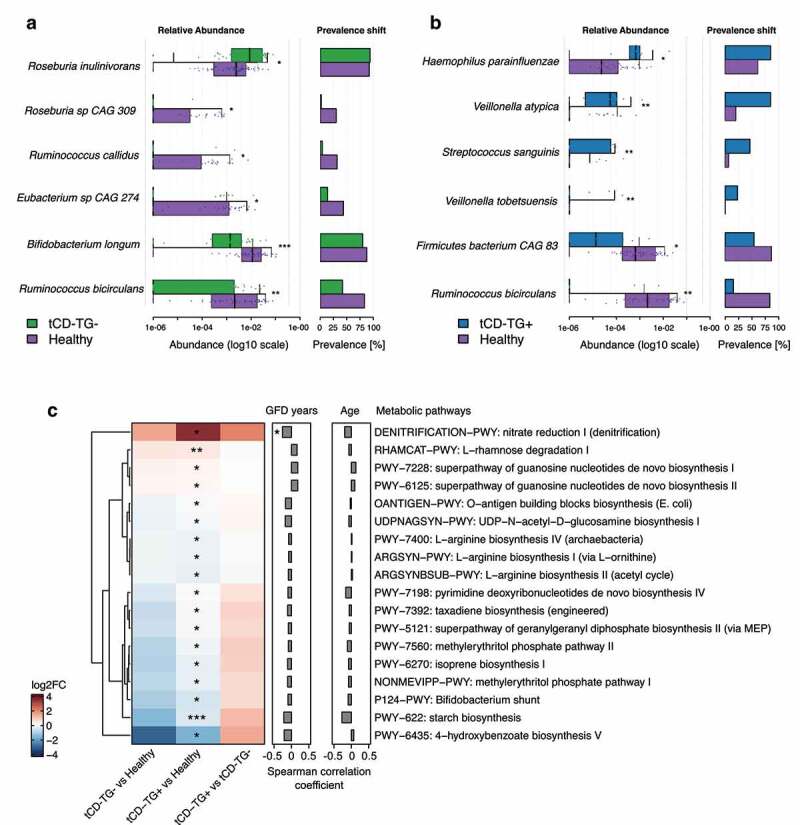
A-B) Plots showing the differentially abundant bacteria by comparing tCD-TG- (a) or tCD-TG+ (b) with healthy controls. For each study group, on the left are reported the microbial relative abundances and on the right the prevalence shift. Wilcoxon Rank-Sum test: *adj. p < .05; **adj. p < .01; ***adj. p < .001. C) Heat map representing the log2FCs of microbial metabolic pathways associated with a significantly different prevalence among the comparisons performed. Wilcoxon Rank-Sum test: *p < .05; **p < .01; ***p < 0.001    . On the right are reported the correlation coefficients computed between the pathway prevalence and the years of GFD or tCD subjects’ age: *p < .05.

At the species level, *Bifidobacterium longum, Roseburia* sp. *CAG 309, Ruminococcus bicirculans, Ruminococcus callidus*, and *Eubacterium* sp. *CAG 274* were less abundant in tCD-TG- with respect to controls (log2FC from −1.74 to −0.51; adj. p < .05) while *Roseburia inulinivorans* resulted more abundance (log2FC = 0.61; adj. p < .05, Figure 3a and Supplementary Table S6c). Conversely, in tCD-TG+, *Veillonella atypica, Veillonella tobetsuensis, Streptococcus sanguinis*, and *Haemophilus parainfluenzae* were more abundant (log2FC from 1.43 to 0.18; adj. p < .05), while *Firmicutes bacterium CAG 83* and *R. bicirculans* were reduced (log2FC of −2.88 and −1.70, respectively; adj. p < .05, Figure 3b and Supplementary Table S6d). No differences were observed between the two tCD groups (Supplementary Table S6e). Microbial abundances and the GFD duration significantly correlated for *H. parainfluenzae* (SCC = −0.31) and *S. sanguinis* (SCC = −0.26) (Supplementary Table S6f), but the latter also correlated with the subject age (SCC = −0.26). The differential abundance of species mentioned above was confirmed by repeating the comparisons with the independent group of controls: all the species reported the same abundance alteration, with six and five of them statistically significant in tCD-TG- and tCD-TG+ compared to controls, respectively (Supplementary Table S6g). In addition, analysis of stool metagenomic profile of the 19 pediatric CD-TG+ patients with respect to healthy controls showed an increase (log2FC = 2.05) of *Veillonella atypica* levels in patient samples, albeit not significant (Supplementary Table S6g).

unctional profiling of gut metagenomes highlighted 18 microbial pathways with a significantly different gene abundance among the groups (Figure 3c and Supplementary Table S6h). Overall, compared with controls, both tCD groups showed a lower abundance in microbial genes involved in the 4-hydroxybenzoate biosynthesis, starch biosynthesis, Bifidobacterium shunt, and amino acids biosynthesis (*L-arginine biosynthesis I, II, and IV)* and a higher abundance in *nitrate reduction and L-rhamnose degradation. Nitrate reduction I* pathway was negatively correlated with the years of GFD (SCC = −0.26) (Figure 3c and Supplementary Table S6f).

### Integration of miRNA, microbiome, and nutrient intake data

**Figure 4.**
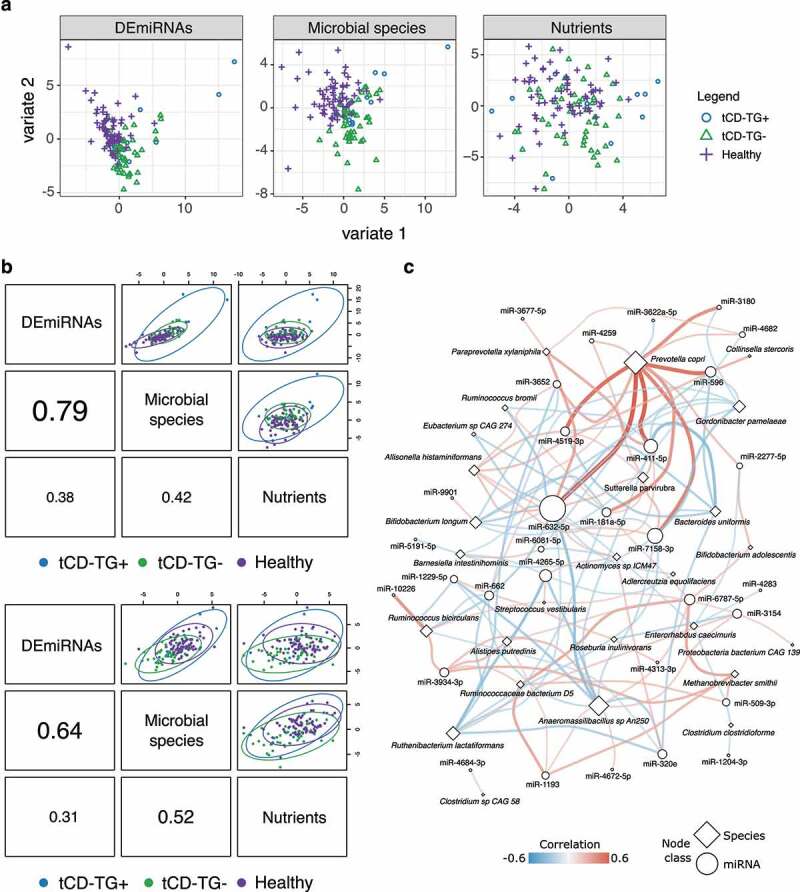
a) Sample plots from DIABLO analysis showing the contribution of stool DEmiRNAs, microbial species, or nutrients in distinguishing both CD categories and controls. The samples are represented as dots plotted according to their projection in the subspace spanned by the latent variables defined by integrating the three data types. b) Correlation between variate 1 (top) and variate 2 (bottom) defined for each data type. c) Network representation of the significant correlations (adj. p < .05) between DEmiRNAs and bacteria identified as discriminating features in the DIABLO analysis. The node size is proportional to their degree, while edge color and width are related to the correlation coefficients.

Integrative analysis with DIABLO showed good efficiency in discriminating both tCD groups from controls based on stool DEmiRNAs and gut microbial profiles, but not nutrient intake (Figure 4a), supported by the high correlation between DEmiRNA and microbial profiles observed for both variates (Figure 4b). Candidate miRNA–microbiome interactions involving the most discriminating miRNAs and microbes (Supplementary Figure S3a) were explored by pairwise correlation analysis (Supplementary Table S6i). Network representation of 121 significant miRNA-microbe correlations (adj. p < .05) highlighted *Prevotella copri* as the bacteria related with the highest number of DEmiRNAs (n = 14), followed by *Anaeromassilibacillus* sp. *An250* (n = 12) and *Ruthenibacterium lactatiformans* (n = 8) (Figure 4c and Supplementary Table S6i). miR-632-5p showed the highest number of related species in the network (n = 16), followed by miR-7158-3p (n = 9), miR-411-5p (n = 8), and miR-4265-5p (n = 7) (Figure 4c and Supplementary Table S6i).

### Identification of features characterizing CD subjects on GFD

Starting from the features selected by the DIABLO analysis, a classification analysis was applied to identify the minimal set with the best classification potential (Supplementary Table S7a). Respectively, 16, 16, and 9 features were able to classify all tCD subjects (Accuracy = 88.8%, AUC = 0.94) tCD-TG- (Accuracy = 87.0%, AUC = 0.96) and tCD-TG+ (Accuracy = 88.2%, AUC = 0.85) with respect to controls (Supplementary Figure S3b upper panels and Supplementary Table S7a,b). Testing the same features between tCD subjects and the independent cohort of controls confirmed their discriminating efficiency (Supplementary Figure S3b lower panels and Supplementary Table S7a).

A subset of features specifically distinguished each of the two tCD groups from controls. miR-1229-5p, *miR-3154*, miR-641, *miR-4313-3p, miR-4672-5p, B. longum, Collinsella stercoris, Eisenbergiella tayi, Enterorhabdus caecimuris, Paraprevotella xylaniphila, R. inulinivorans, Veillonella dispar*, and the PUFA intake were the features pertaining the classification between tCD-TG- and controls. Conversely, *miR-3180, miR-662, Anaeromassilibacillus* sp. *An250 Allisonella histaminiformans*, and the total saturated fatty-acids and linoleic acid intake characterized the signature discriminating tCD-TG+ from controls. *R. bicirculans*, vitamin E, and total lipid intake were the features of both identified feature sets (Supplementary Table S7b).

## Discussion

This study represents the first characterization of stool sncRNAs in CD patients on GFD with a concomitant description of their gut microbiota. For several miRNAs, a differential expression was observed in tCD-TG- and tCD-TG+ subjects with respect to controls negative for TG antibody levels. In tCD-TG+, some miRNAs were dysregulated both in comparison with the controls and, following the same trend, with tCD-TG-. This aspect may be ascribed to the fact that tCD-TG+ reported gastrointestinal symptoms and tested positive for TG antibody levels due either to an incorrect or a very recent adherence to the GFD. One of the well-known effects of GFD in CD patients is the reduction of duodenal inflammation. In the present research, coherently, the targets of DEmiRNAs up-regulated in tCD-TG+ were involved in inflammatory-response-related pathways, which are widely associated with CD.^1^ Among them, miR-148-3p was previously reported to regulate the inflammatory dendritic cell differentiation in autoimmune disorders^30^ and macrophage differentiation,^31^ as well as inhibit gut inflammation in a mouse model of colitis.^32^ Instead, miR-181b-5p, another miRNA up-regulated in tCD-TG+, was described as an effector of Signal transducer and activator of transcription 3 (STAT3) and NF-κB signaling.^33,34^ The altered expression of these inflammation-related miRNAs in stool of tCD-TG+ individuals could be explained by an inflammatory response triggered by the gluten ingestion.^35^ Similarly, the targets of the DEmiRNAs down-regulated in tCD-TG+ were involved in the activation of eIF2α/PERK axis in response to endoplasmic reticulum stress, a process previously described in autoimmune diseases,^36,37^ including CD.^38^

Besides reflecting changes in the inflammatory state of the intestinal tissues, the observed stool DEmiRNAs could mirror epigenetic modifications in gut cells driven by a long-term dietary regime. Interestingly, miR-638-3p, significantly more abundant in tCD-TG- subjects, was among the most significantly represented in feces from vegan/vegetarian subjects in Tarallo *et al*.^16^ miR-638 was previously related to CD, where its overexpression was detected in duodenal biopsies.^39^ Notably, the TG-coding gene (*TGM2*) is among the predicted targets of this miRNA. Other eight DEmiRNAs (miR-2681-3p, miR-599, miR-423-5p, miR-6832-5p, miR-12120, *miR-4313-3p*, miR-1291, and *miR-6081*-5p), more abundant in tCD-TG- compared to tCD-TG+ and controls, could represent promising molecules to investigate the long-term effect of GFD. Several miRNAs correlated with the GFD length irrespectively of subjects’ age, including the previously mentioned miR-2681-3p. These results further support the role of diet on the modulation of miRNA expression over time, as previously observed.^16^ As an additional interesting aspect, compared to subjects who recently started the GFD, those with a longer adherence showed levels of miR-4533-3p and miR-2681-3p fairly similar to controls, suggesting a possible recovery to a physiological level of the expression of these miRNAs over the years. Although more studies are needed to elucidate the molecular activity of these miRNAs, our findings provide evidence of the role of long-term dietary changes in reprogramming the post-transcriptional regulatory network of intestinal cells, which is reflected in stool samples. However, to better understand how GFD affects stool miRNA expression, it would also be pivotal to investigate their profiles in healthy subjects on GFD.

Stool levels of seven miRNAs were coherently significantly altered in both tCD-TG- and tCD-TG+. These overlapping DEmiRNAs may be related to genetic/epigenetic changes associated with the pathology itself and not necessarily to the GFD adherence. The functional analysis showed that the target genes of these DEmiRNAs are involved in the integrin-mediated signaling pathways (Supplementary Table S4a). The key role of integrins in triggering immune mechanisms has been largely described, even if their involvement in the onset and progression of autoimmune diseases (including CD) remains elusive.^40^ This set of miRNAs could have potential future applications in clinical trials for the diagnosis and monitoring of CD as well as therapeutic targets to reduce villous atrophy.

Stool profiling of other sncRNAs besides miRNAs highlighted altered levels of some piRNAs and tRNAs in tCD subjects, with a marked up-regulation in tCD-TG+. This novel finding suggests that such molecules could have an unexplored role in the regulatory networks associated with CD and their expression may be modulated by GFD. The role of sncRNAs other than miRNAs has been scarcely investigated^41^ and, especially for piRNAs, their sequences are sometimes similar and highly repeated in the human genome, making their functional characterization non-trivial. Further analyses are needed to clarify the role of these molecules in relation to CD and GFD.

In the present study, exploring the gut microbiome composition at phylum level, we confirmed a reduction in Actinobacteria and Verrucomicrobia and an increase in Bacteroidetes abundances in tCD-TG-subjects, while Euryarchaeota and Fusobacteria were specifically reduced in tCD-TG+.^42,43^ At species levels, a decrease of *B. longum* was observed in both tCD groups, coherently with previous observations.^44,45^ Interestingly, a specific strain of this bacterium, namely *B. longum* CECT 7347, was observed to reduce the production of inflammatory cytokines, CD4+ T cells and peripheral CD3+ T lymphocytes^46^ and its oral administration ameliorated the enteropathy induced by gliadin ingestion.^47^ In addition, *S. sanguinis*, more abundant in tCD-TG+, was also increased in the saliva of CD patients compared to controls.^48^ Interestingly, *S. sanguinis* and *H. parainfluenzae* were negatively correlated with the GFD years, supporting that the GFD adherence could restore a microbial abundance comparable to that of healthy controls.

Microbial functional profiling revealed in tCD-TG- a lower abundance of genes involved in starch metabolism. This is in line with the adherence to GFD, which includes gluten-free products characterized by a different content of starch and carbohydrates.^49^ This aspect was also supported by the nutrient intake data showing a lower starch intake in tCD-TG- individuals with respect to controls (Supplementary Table S1). Conversely, the abundance of genes involved in the nitrate reduction process was increased in tCD-TG+ and (although not significantly) also in tCD-TG- group with respect to healthy controls, and this metabolic pathway was negatively correlated with the years of GFD. This finding is consistent with previous observations on the reduction of nitrate catabolites, particularly nitric oxide (NO), in duodenal tissue, urine, and plasma of CD patients on a GFD.^50^ Indeed, NO is a well-known biomarker of intestinal inflammation,^51^ and the reduction of microbial taxa annotated with the nitrate reduction pathway might be related to the decrease in duodenal inflammation related to the GFD.

The observed alterations in miRNA and microbial profiles may also be due to an effect of the specific nutrient intake. Although no single correlation between the DEmiRNA levels/microbial species abundances and the nutrient intakes reached the statistical significance after adjustment for multiple tests (Supplementary Table S1c), some of these correlations may deserve further investigation. This includes a moderate positive correlation between linolenic acid and miR-635-3p levels and negative correlations between folic acid and *miR-4309-3p*, as well as between Vitamin E and miR-596. For microbial species and nutrient intake, the most relevant but still not significant correlation was observed between *R. inulinivorans* and vitamin C and beta-carotene. Given these results, we can conclude that no single nutrient strongly influenced the study outcomes, accordingly with the limited differences observed in the dietary pattern between tCD-TG- and healthy subjects. However, we cannot rule out a possible cumulative effect due to the small effects of the variegated intake of multiple nutrients.

In contrast, the observed correlations among miRNAs and microbes altered in tCD subjects provide evidence of candidate host-microbial interactions in this disease. For instance, *P. copri*, even if not significantly differentially abundant among the analyzed groups, was related to the highest number of DEmiRNAs, followed by *R. bicirculans. P. copri* abundances progressively decreased in stool from tCD-TG+ and tCD-TG- to controls. Conversely, *R. bicirculans* had an opposite trend with increasing abundances going from tCD-TG+ to controls. Although the literature concerning this bacterium is still scarce, a recent study supported its ability to utilize certain hemicelluloses, especially β-glucans and xyloglucan for its growth.^52^ In addition, an increased abundance of this bacterium has been reported in relation to a high-protein low-fat weight reduction diet^53^ and a low calorie weight loss diet supplemented with resistant starch.^54^ This is in line with the previously mentioned lower starch intake and related metabolism observed for the tCD groups, which could partially explain its reduction in those categories. Moreover, *R. bicirculans* lower abundance was recently associated with pulmonary arterial hypertension severity^55^ and alopecia areata.^56^ Considering its abundance reduction, especially in the tCD-TG+, its possible involvement in inflammatory/autoimmune processes cannot be excluded. Interestingly, a high abundance of *Prevotella* and a low representation of the *Ruminococcus* genus were observed in stool samples of a *Rhesus macaque* model of CD.^57^ In the same study, inflammation-related miRNAs were up-regulated in the jejunum of gluten-sensitive animals and some of them were predicted to interact with bacterial 16S. However, despite evidence emerging on functional interactions between host small RNAs and microbial genes,^22^ further analyses are needed to clarify the miRNA-bacteria relations observed in our research.

The integration of miRNome, microbiome, and daily nutrient intake data showed the ability to discriminate CD categories from controls, with the major contribution provided by specific DEmiRNAs and microbial species. Nutrient intake data alone could not distinguish the analyzed groups, as expected, given that all the CD subjects were on GFD and limited differences were observed in the nutrient intake. Interestingly, only a small overlap was observed comparing the signature of features distinguishing tCD-TG- or tCD-TG+ with respect to controls (except for Vitamin E and *R. bicirculans*). This suggests that the identified markers may precisely characterize the effects of long-term GFD adherence or a perturbation related to a gut inflammatory state.

Despite the promising results, a limitation of the present study was the small number of tCD-TG+ and untreated CD-TG+ adult individuals recruited. Indeed, although in the latter group we observed coherent alterations with tCD-TG+ for some of the identified DEmiRNAs as well as microbial relative abundances, we could not analyze these subjects as an independent group, due to its limited sample size. However, we tried to overcome this limitation by analyzing a pilot dataset from pediatric untreated CD-TG+ subjects, in which most DEmiRNA alterations were also confirmed.

Conversely, this work presents various strengths: 1) being the first observational study using a miRNome-wide approach on fecal samples of CD patients; 2) among the few investigations assessing gut microbiome composition by shotgun metagenomic sequencing in relation to the disease;^45^ 3) correlating microbial relative abundances and host miRNome profiles from the same specimens of the same subjects; 4) including a healthy control group recruited and analyzed in concomitance with CD subjects, matched for age and gender and with negative TG serology; and, finally, 5) providing the validation of the observed miRNA and microbiome alterations by comparing findings with an independent group of healthy controls, or, for stool miRNAs alone, with a group of newly diagnosed untreated CD-TG+ children.

In conclusion, we showed the modulation of several human miRNAs in relation to GFD adherence in CD subjects which can be assessed in fecal samples. Interestingly, the expression levels of some of these miRNAs, over the years of the dietary regime, show reduced differences with respect to healthy subjects. Conversely, the short/not strict adherence to GFD confirmed molecular alterations of inflammatory pathways related to CD. Similarly, the microbiome composition reflected the active status of the disease or GFD compliance, confirming previous findings in the literature. Finally, as a novel finding, the integrated analysis of fecal miRNAs and gut microbial species supports the host-microbiota crosstalk in physiological and pathological conditions and may provide composite noninvasive molecular biomarkers for GFD adherence monitoring.

## Supplementary Material

Supplemental Material

## Data Availability

Relevant data presented in the study are included in the article or uploaded as supplementary information. Sequencing raw data are available in the indicated repositories, while metadata are available upon request to the corresponding author.
