# Supplementary material for: Gluten-free diet affects fecal small non-coding RNA profiles and microbiome composition in celiac disease supporting a host-gut microbiota crosstalk
Source: Gut Microbes. 2023 Feb 7;15(1):2172955. doi: 10.1080/19490976.2023.2172955 (PMC9928459; doi:10.1080/19490976.2023.2172955)
Supplement: Supplemental Material [file KGMI_A_2172955_SM4401.zip › Supplementary Materials_Revised.docx]

**Supplementary materials and methods**

**Sample collection**

Blood was collected according to standard phlebotomy procedures when volunteers returned the stool samples to the laboratory or, in case of those subjects recruited at the hospital, the day of their gastroenterological visit. Blood samples were collected into Ethylenediaminetetraacetic acid (EDTA) and serum clot activator tubes and processed for plasma and serum separation, aliquoted and stored at -80ºC within 2 hours from sampling as described in Tarallo et al.[1].

Stool samples were collected in collection tubes with nucleic acids stabilizing solution (Norgen Biotek). The returned samples were resuspended within a few hours by vortex and distributed in aliquots (approximately 200µl each) stored at -80°C.

**Small RNA sequencing**

Small RNA transcripts were converted into barcoded cDNA libraries using a NEBNext multiplex small RNA library prep set for Illumina (protocol E7330; New England BioLabs). For each sample, 250ng of RNA were used for the library preparation which was performed with a unique indexed primer to allow libraries pooling into one sequencing lane. This procedure has been previously described in Tarallo et al. [1].

The libraries (24 samples multiplex) were subjected to the Illumina sequencing pipeline, passing through clonal cluster generation on a single-read flow cell (Illumina) by bridge amplification on a cBot and 75 sequencing cycles using a HiSeq 4000 (Illumina) at the Gene Core Facility of the European Molecular Biology Laboratory (EMBL), Heidelberg (Germany).

The small RNA-sequencing analyses were performed according to [2]. A miRNA was considered detected if supported by more than 15 normalized reads in at least one sample. Functional enrichment analysis was performed using RBiomirGS v0.2.12[3] considering validated miRNA-targets interactions and using as input the average log2FC and the combined adjusted p-value computed between tCD groups and healthy. Gene sets characterized by an adjusted p-value <0.05 were considered as enriched. Semantic similarity analysis was performed using *rrvgo* v1.2.0 R package on the list of GO Biological Processes terms enriched by the RBiomirGS analysis. The similarity was computed using the Resnik method and considering a similarity threshold of 0.75.

**Shotgun metagenomics sequencing**

Taxonomic profiling was performed with MetaPhlAn3 in default settings with mpa_v30_CHOCOPhlAn_201901 as markers database. Diversity metrics were computed using the vegan R package. Functional profiles were obtained by HUMAnN 3.0 in default settings [4]. Differences in microbial relative abundances were tested using Wilcoxon Rank- Sum test and SIAMCAT v1.11.1 in default settings [5]. Species with an abundance lower than 0.001 were filtered out using the filter.features function.

**Statistical and computational analyses**

Statistical analyses were performed with R v4.0.4. Age and sex-adjusted differential expression analysis was performed with DESeq2 v1.22.2 (LRT function) [6]. A miRNA/sncRNA was considered differentially expressed (DEmiRNA/DEsncRNA) if associated with a Benjamini-Hochberg (BH)-adjusted p<0.05 and a median number of normalized reads >15 in at least one group. Correlation analyses between miRNA levels and GFD years or subjects’ age were performed using the Spearman’s rank correlation coefficient (SCC) and correlations with a p<0.05 were considered significant. SCC was also computed between levels of each miRNA-bacteria pair (adjusted for multiple-testing correction with the BH method). Analysis of monotonic miRNA expression trend was performed using the Mann-Kendall test. A miRNA was considered characterized by a trend if associated with a p<0.05 and with median levels progressively increasing/decreasing from untreated CD to tCD-TG+, tCD, and healthy subjects. All correlation analyses were performed using the cor.test function in R.

DIABLO module of MixOmics v.6.14.1 was used to integrate taxonomic profiles, DEmiRNA levels, and dietary information[7]. Datasets were pre-processed with nearZeroVar R functions. The top 25 features with the highest loading on DIABLO components were used as input to classify CD groups and healthy subjects using the Random Forest classifier of Weka v.3.8.5 (https://www.cs.waikato.ac.nz/ml/weka/) and a 10-Fold Cross-Validation approach. Weka CfsSubsetEval module was applied in default settings for the feature selection.

**Supplementary Figures**

**Supplementary Fig. 1. A)** Box plot reporting the stool levels of miR-4533-3p and miR-2681-3p in tCD-TG- stratified by GFD length and in healthy controls. **B)** Box plots reporting the normalized levels of nine stool DEmiRNAs characterized by an increasing/decreasing expression change in samples from untreated CD-TG+ to healthy subjects. **C**) Scatter plot reporting the log2FC computed between adult tCD-TG+ (x-axis) or untreated paediatric CD-TG+ subjects (y-axis) when compared to matched healthy controls. The results of 30 DEmiRNAs characterized by a coherent differential expression is reported. **D)** Heatmap showing for the 30 DEmiRNAs of panel C, the log2FC from the differential expression analysis between: tCD-TG+ and tCD-TG- with respect to healthy controls (1^st^ and 5^th^ columns); tCD-TG+ with respect to an independent group of healthy subjects (2^nd^ column); tCG-TG+ including three untreated adult CD-TG+ subjects with respect to healthy controls (3^rd^ column); and the comparison between untreated paediatric CD-TG+ patients and matched healthy controls (4^th^ column).

**Supplementary Fig. 2.** Heatmap reporting the log2FCs of the differentially expressed sncRNAs (DEsncRNAs) other than miRNAs computed in each comparison.

**Supplementary Fig. 3. A)** Bar plot showing the loading weights of each DIABLO-selected variable for variate 1 (top panels) and variate 2 (bottom panels). The colour indicates the study group in which the variable was characterized by the highest median level. **B)** Receiver Operating Characteristic (ROC) curve for the classification of healthy subjects with respect to all CD subjects together, tCD-TG-, or tCD-TG+ based on the levels of discriminating features identified by DIABLO and Weka analysis. Analysis performed with respect to the healthy group of this study (Comparison 1) are reported at the top, while on the bottom those from the classification considering omnivorous subjects from [1] (Comparison 2). The Area Under the Curve (AUC) computed in each analysis is reported.

**Legend of the Supplementary Tables**

**Supplementary Table 1.** <https://figshare.com/s/fab15b4aba96afa80d3d>

**A**) Clinical and anamnestic information of the study cohorts. **B**) Intake of foods, drinks, and dietary nutrients as estimated by the EPIC questionaries for each study group. The log2FC of intake between the study groups is reported the result of the statistical analysis as well (Wilcoxon Rank-Sum test and Kruskal-Wallis’s test). **C)** Results of the Spearman correlation analysis between the estimated nutrient intakes and the levels of miRNAs and species identified as differentially abundant among the study groups.

**Supplementary Table 2**. <https://figshare.com/s/7afbc83ba9c6c80f1a2f>

**A)** Statistics of quality control and alignment of the sRNA-Seq reads. **B)** Statistics of the pre-processing and alignment of shotgun metagenomic sequencing reads.

**Supplementary Table 3**. <https://figshare.com/s/724c3b3e6ded1264c40d>

**A**) Characteristics of the study cohorts. **B**) Results of the stool miRNA differential expression analysis with DESeq2. **C**) Analysis of DEmiRNAs expression changes considering an independent group of healthy individuals. H, healthy from this study; H2, healthy omnivorous from Tarallo et al., 2021. **D**) Results of the Spearman correlation analysis between DEmiRNA stool levels and the years of GFD or the subject’s age. **E**) Analysis of DEmiRNAs expression changes considering a cohort of untreated paediatric CD-TG+ patients matched with healthy controls.

**Supplementary Table 4**. <https://figshare.com/s/71e2b1cfbaac09438aba>

**A**) Results of the functional enrichment analysis of DEmiRNA target genes. For the Gene Ontology Biological Processes terms, the results from the semantic similarity are also reported. **B**) Validated miRNA-target interactions used for the enrichment analysis. **C**) List of miRNA-target interactions supported by the highest number of evidence.

**Supplementary Table 5**. <https://doi.org/10.6084/m9.figshare.19229880>

**A**) Results of the DESeq2 differential expression analysis of sncRNA stool levels. **B**) Analysis of DEmiRNAs expression changes considering an independent group of healthy individuals. H, healthy from this study; H2, healthy omnivorous from Tarallo et al., 2021.

**Supplementary Table 6**. <https://figshare.com/s/97cd345bdd3b04f23bee>

**A**) Alpha diversity values computed for each sample. **B**). Description and differential analysis of the abundances of microbial phyla. **C-E**) Results of the SIAMACAT analysis for **C**) tCD vs Healthy, **D**) tCD-TG+ vs Healthy, and **E**) tCD-TG+ vs tCD. **F**) Evaluation of the abundances of species identified as significantly different by SIAMCAT considering an independent cohort of healthy controls and data of paediatric CD-TG+ patients. **G**) Results of the correlation analysis between the levels of microbial species and signalling pathways identified as significantly differentially prevalent among the study groups. **H**) Results of the differential pathway analysis performed among the study groups. **I**) Result of the correlation analysis between microbial abundances and the stool DEmiRNA levels.

**Supplementary Table 7**. <https://figshare.com/s/9208c8981afa72c7c56f>

**A**) Results of the Weka classification analysis using all features identified by DIABLO or only the subset contributing more to the classification. The analysis was performed considering the healthy control of this study or the healthy omnivorous subjects from Tarallo et al., 2021 (Healthy2). **B**) The list of features contributing more to classify the different study groups.

**Supplementary Material References**

1. Tarallo S, Ferrero G, De Filippis F, et al. Stool microRNA profiles reflect different dietary and gut microbiome patterns in healthy individuals. Gut 2021.
2. Tarallo S, Ferrero G, Gallo G, et al. Altered Fecal Small RNA Profiles in Colorectal Cancer Reflect Gut Microbiome Composition in Stool Samples. mSystems 2019;4.
3. Zhang J, Storey KB. RBiomirGS: an all-in-one miRNA gene set analysis solution featuring target mRNA mapping and expression profile integration. PeerJ 2018;6:e4262.
4. Franzosa EA, McIver LJ, Rahnavard G, et al. Species-level functional profiling of metagenomes and metatranscriptomes. Nat Methods 2018;15:962-968.
5. Wirbel J, Pyl PT, Kartal E, et al. Meta-analysis of fecal metagenomes reveals global microbial signatures that are specific for colorectal cancer. Nature medicine 2019;25:679-689.
6. Love MI, Huber W, Anders S. Moderated estimation of fold change and dispersion for RNA-seq data with DESeq2. Genome Biol 2014;15:550.
7. Singh A, Shannon CP, Gautier B, Rohart F, Vacher M, Tebbutt SJ, et al. DIABLO: an integrative approach for identifying key molecular drivers from multi-omics assays. Bioinformatics 2019;35:3055-62.
