# Supplementary figures and images for: Gluten-free diet affects fecal small non-coding RNA profiles and microbiome composition in celiac disease supporting a host-gut microbiota crosstalk
Source: Gut Microbes. 2023 Feb 7;15(1):2172955. doi: 10.1080/19490976.2023.2172955 (PMC9928459; doi:10.1080/19490976.2023.2172955)

**A**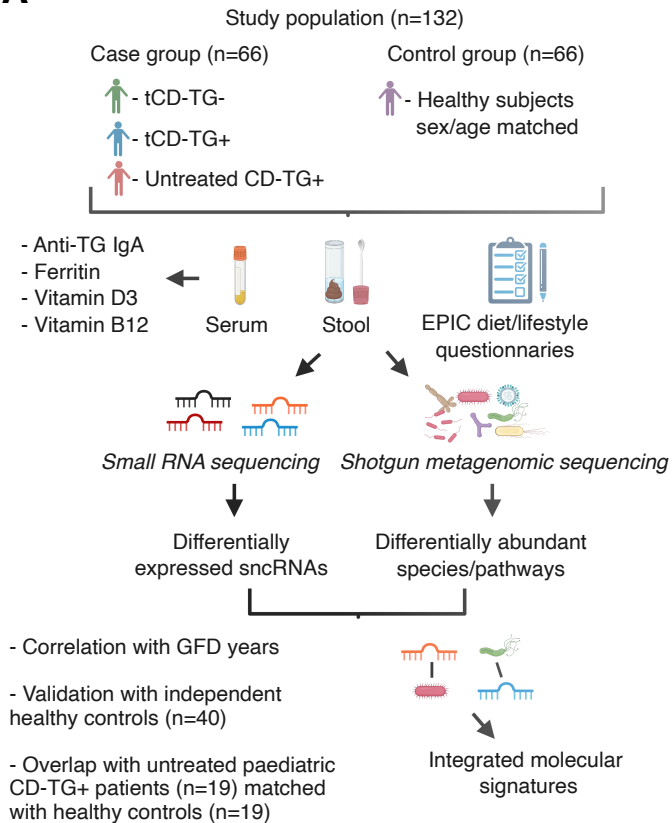**B**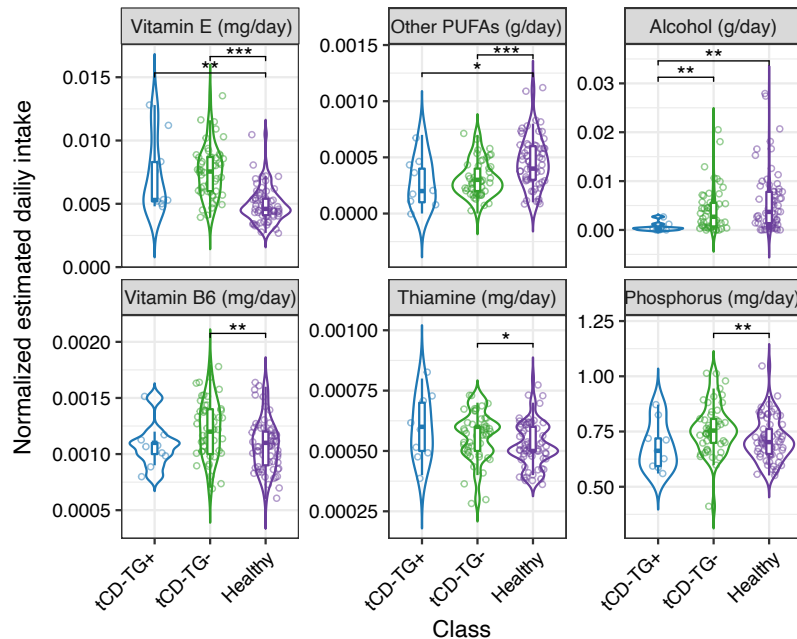

Supplement: Supplemental Material [file KGMI_A_2172955_SM4401.zip › Supplementary_Figure_1.pdf]

**A**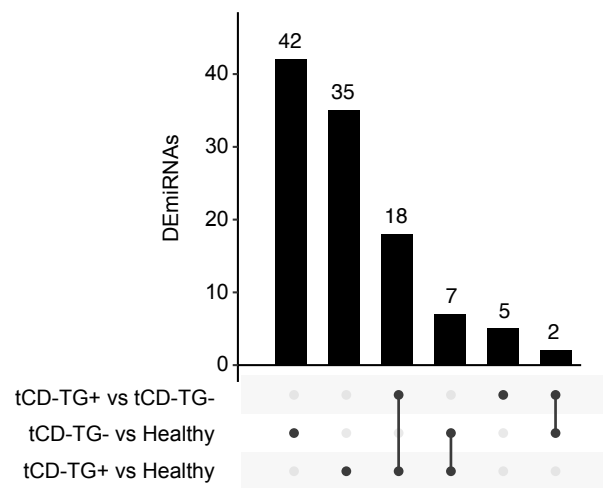**B**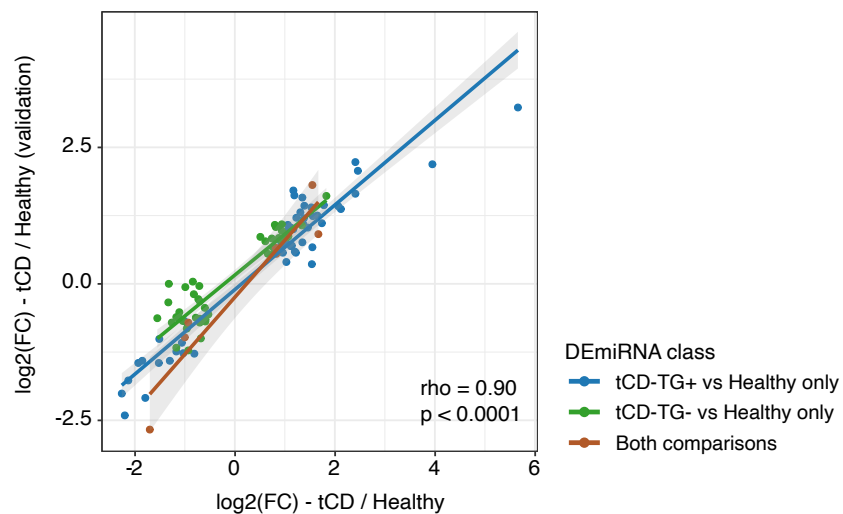**C**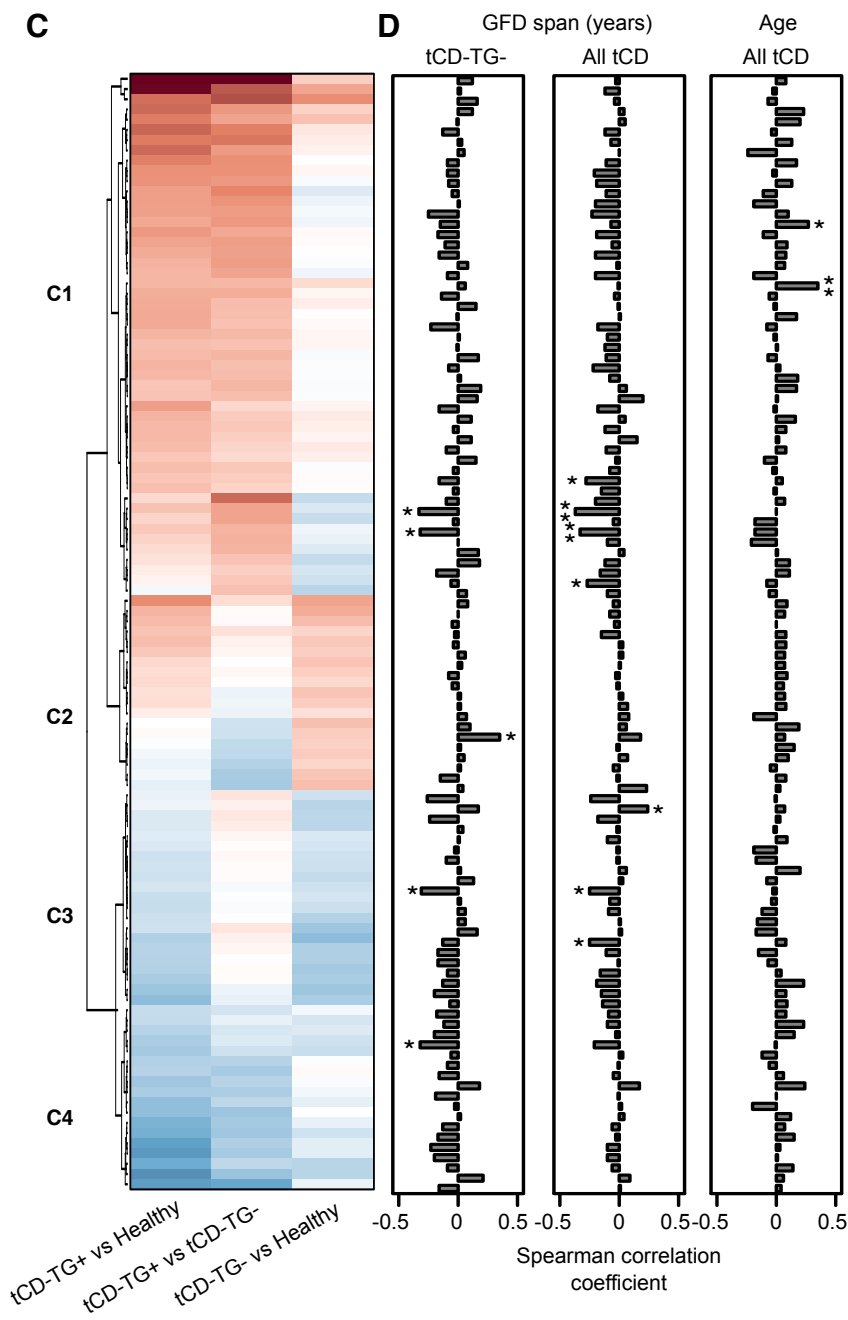**E**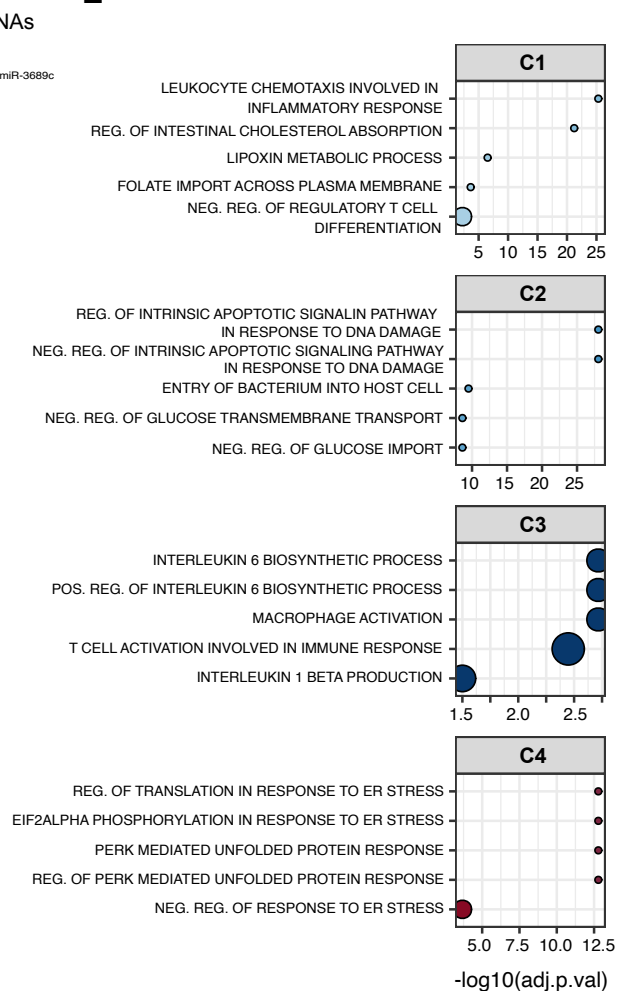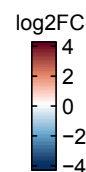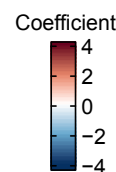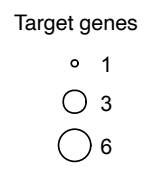

Supplement: Supplemental Material [file KGMI_A_2172955_SM4401.zip › Supplementary_Figure_2.pdf]

A

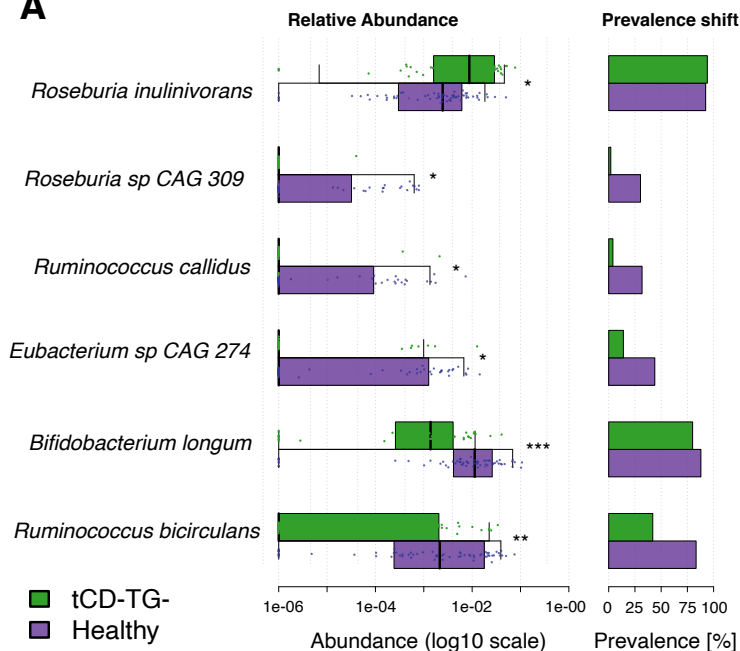

B

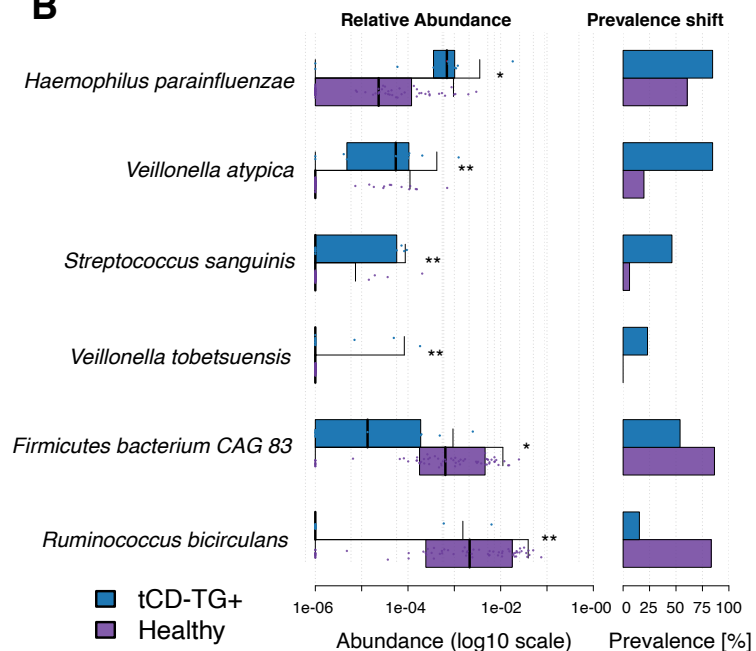

C

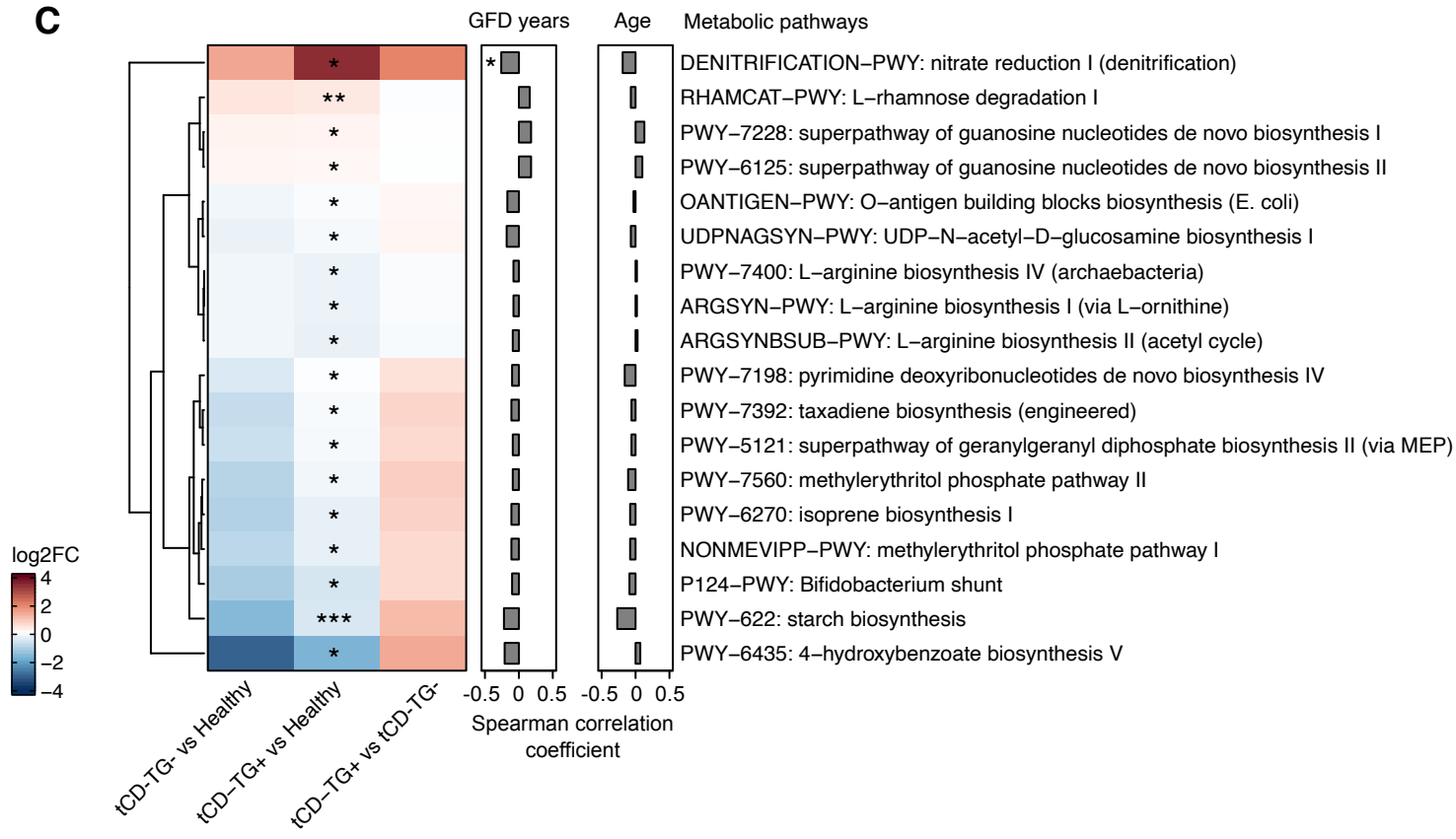

Supplement: Supplemental Material [file KGMI_A_2172955_SM4401.zip › Supplementary_Figure_3.pdf]
